# Notes from Beethoven’s genome

**DOI:** 10.1016/j.cub.2024.01.025

**Published:** 2024-03-25

**Authors:** Laura W. Wesseldijk, Tara L. Henechowicz, David J. Baker, Giacomo Bignardi, Robert Karlsson, Reyna L. Gordon, Miriam A. Mosing, Fredrik Ullén, Simon E. Fisher

**Affiliations:** 1 Department of Neuroscience, Karolinska Institutet, Stockholm, Sweden.; 2 Department of Psychiatry, Amsterdam UMC, University of Amsterdam, Amsterdam, The Netherlands.; 3 Department of Cognitive Neuropsychology, Max Planck Institute for Empirical Aesthetics, Frankfurt am Main, Germany.; 4 Music and Health Sciences Research Collaboratory, Faculty of Music, University of Toronto, Toronto, Canada.; 5 Krembil Centre for Neuroinformatics, Centre for Addiction and Mental Health, Toronto, Canada.; 6 Institute for Logic, Language, and Computation, University of Amsterdam, Amsterdam, The Netherlands.; 7 Language and Genetics Department, Max Planck Institute for Psycholinguistics, Nijmegen, The Netherlands.; 8 Max Planck School of Cognition, Leipzig, Germany.; 9 Department of Medical Epidemiology and Biostatistics, Karolinska Institutet, Stockholm, Sweden.; 10 Department of Otolaryngology — Head and Neck Surgery, Vanderbilt University Medical Center, Nashville, TN, USA.; 11 Blair School of Music, Vanderbilt University, Nashville, TN, USA.; 12 Vanderbilt Genetics Institute, Vanderbilt University, Nashville, TN, USA.; 13 Department of Hearing and Speech Sciences, Vanderbilt University Medical Center, Nashville, TN, USA.; 14 Melbourne School of Psychological Sciences, Faculty of Medicine, Dentistry, and Health Sciences, University of Melbourne, Melbourne, Australia.; 15 Donders Institute for Brain, Cognition and Behaviour, Radboud University, Nijmegen, The Netherlands.; 16 Senior author.

Rapid advances over the last decade in DNA sequencing and statistical genetics enable us to investigate the genomic makeup of individuals throughout history. In a recent notable study, Begg *et al*.^1^ used Ludwig van Beethoven’s hair strands for genome sequencing and explored genetic predispositions for some of his documented medical issues. Given that it was arguably Beethoven’s skills as a musician and composer that made him an iconic figure in Western culture, we here extend the approach and apply it to musicality. We use this as an example to illustrate the broader challenges of individual-level genetic predictions.

When the societal impact of a renowned figure relates to their exceptional talent or expertise in a certain domain, like music, a commonly asked question is: to what extent is their exceptional performance influenced by genetics? This old line of inquiry, dating back to the earliest days of human genetics^2^, may now appear more addressable due to modern molecular methods. But how reliable are the resulting answers given our current state of knowledge?

Begg *et al.*^1^ focused on possible genetic predispositions to Beethoven’s health ailments. To investigate this, they largely relied on polygenic indices (PGIs), which represent an individual’s genetic propensity for a specific trait, informed by the estimated effects of common DNA variants (typically single-nucleotide polymorphisms, or SNPs) derived from a prior genome-wide association study (GWAS) on the trait in question^3^. Such a PGI reflects the aggregate influence of a great many SNPs spread through the genome, each with individually tiny effect sizes, but captures only a fraction of genetic variance overall (at least for most complex traits). Nevertheless, Begg *et al.* found Beethoven’s PGI for liver cirrhosis at the 96^th^ percentile, and suggested that genetic factors may have contributed to his well-known severe liver disease, over and above effects of heavy drinking and hepatitis.

Begg *et al.*^1^ did not address Beethoven’s most famed traits, likely because there has not yet been a sufficiently informative GWAS of musical talent. However, in a recent GWAS involving 606,825 individuals with European ancestry, 69 genetic loci were significantly associated with variation in self-reported beat synchronization ability, assessed with the question: ‘Can you clap in time with a musical beat?’^4^. The variance explained by the aggregate of all common genetic variants across the genome (SNP-based heritability) was 13–16%. Although self-report on a single item is limited as a measure of musical talent, the study found PGIs based on this GWAS to predict musicianship status (R^2^ = 2%) and a follow-up family study showed that the PGIs tapped into broader genetic musical propensity, with small but significant effects on an array of music-related skills^5^ (R^2^ between 1–3%, in line with other behavioural traits^3,6^).

Here, we calculated this PGI for Beethoven and compared it with two population-based datasets of thousands of modern-day individuals for whom we have musical achievement data. As emphasized in our preregistration (https://aspredicted.org/fj2cu.pdf), we made no prior prediction as to where Beethoven’s PGI would fall, since we intended this as an illustration of the limitations of the approach. We leveraged genotype data from 8,344 individuals (5,648 with musical achievement data) from the Swedish Twin Registry’s STAGE cohort^7^, 6,150 individuals from Vanderbilt’s BioVU cohort^4^, and genome sequence data from Beethoven^1^. Supplementary material gives more information on samples, phenotype definitions, quality control, and data processing.

Beethoven’s PGI for beat synchronization places him in the 9th percentile for STAGE (Figure 1A) and the 11th percentile for BioVU (Figure 1B), indicating a relatively low PGI compared with both reference cohorts. Comparison of Beethoven’s data with the cohorts’ principal components (Figure S1E,F) suggests that the results are unlikely to be influenced by ancestry differences.

At first glance, the pattern of results may seem somewhat puzzling. On the one hand, this PGI (as in prior work) appears predictive of musical achievement and musicianship status (Figure 1C,D). On the other, Beethoven, one of the most celebrated musicians in history, scored unremarkably, ranking between the 9^th^ and 11^th^ percentile based on modern samples. Yet, such a discrepancy is not that unexpected.

First, a typical PGI captures just a fraction of genetic effects, including only common and no rare DNA variants, and predictive ability is heavily reliant on the statistical power of the discovery GWAS and the heritability of the target trait (in twin studies music-related traits have shown average heritability of 42%^8^). The predictive value of PGIs improves with larger GWAS samples and for traits with higher heritability^6^. Second, genetic associations reflect a culture-specific interplay between underlying heritable factors and environmental influences^3^. Findings from a GWAS in modern Western society may not apply universally across time and regions. Third, as population-level approximations, PGIs do not necessarily yield accurate predictions at the level of the individual^9^. Inevitably many individuals will score high on such a PGI yet low on its target trait, and vice versa. Figure 1C,D illustrates the large variability and overlap in PGIs across different levels of musical achievement and engagement.

Beyond these general issues with PGIs, the findings here might relate to the particular choice of trait, constrained by the current availability of just one well-powered GWAS in this field. Musicality is not a single trait but is better conceptualized as a multicomponent suite of skills that involves a mixture of genetic contributions, some shared across traits, others uniquely influencing particular aspects, limiting the informativeness of any single PGI. Here, the musical trait in the discovery GWAS (self-report of ability to clap to a musical beat) is unlikely to discriminate well in the high range of musical creativity, since a small proportion of people respond negatively to this question. Hence, it is possible that results would have been different for a PGI of a GWAS trait more able to capture variance in the high-range of musical creativity. The Musicality Genomics Consortium (https://www.mcg.uva.nl/musicgens/) is conducting meta-GWASs on a variety of different musical abilities. Above all, it is essential to keep in mind that human traits, including musical skills, are not determined solely by genes or environment, but rather shaped by their complex interplay, and that genetic influences, such as those captured by PGIs, are probabilistic rather than deterministic causes that shape an individual’s future^9,10^.

Analysing the DNA of Beethoven, an individual who lived over two centuries ago, distinctly highlights the challenges of PGI approaches. Obviously, it would be wrong to conclude from the pattern of PGI findings that Beethoven’s musical abilities were unexceptional. Caution is needed when utilizing PGIs for individual-level prediction, including for historical/famous figures, just as for any other factor with low predictive value. Despite such challenges, PGIs are useful for group-level analyses, as research tools for gaining insights into genetic risks over a lifetime and their interplay with the environment.

## Supplementary Material

MMC1

## Figures and Tables

**Figure 1.**
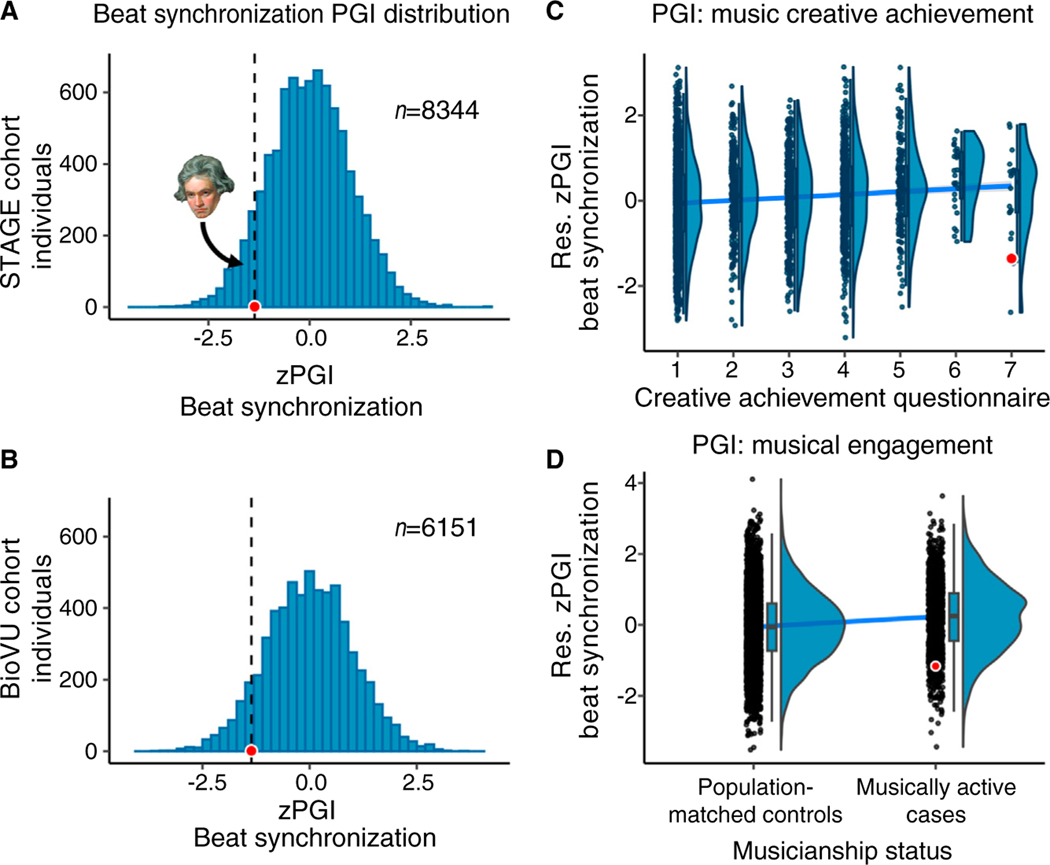
Beethoven’s beat synchronization PGI ranks between the 9^th^ and 11^th^ percentile of modern samples’ beat synchronization PGI. The black dashed line and red dot represent Beethoven’s PGI with respect to (A) STAGE and (B) BioVU cohorts. Raincloud plots depicting the relationship between the beat synchronization residualized (res.) PGI and musical achievement (C) and musical engagement in BioVU (D). We first regressed the first 10 PCs from the PGIs and used the residuals for illustrative and analytical purposes. Dots represent STAGE individual scores, with Beethoven’s PGI in red; the oblique line represents the line of best fit, which was calculated excluding Beethoven’s PGI. Details in Supplemental information. STAGE: Screening Twin Adults: Genes and Environment Swedish Twin Registry; BioVU: Vanderbilt Biorepository; PC: Principal Component; Res.: Residualized. Ludwig van Beethoven image in panel A was adapted from https://commons.wikimedia.org/wiki/File:Beethoven.jpg, by J. K. Stieler, *Portrait Beethovens mit der Partitur zur Missa Solemnis*, 1820. Public Domain.
