# Supplementary material for: Notes from Beethoven’s genome
Source: Curr Biol. Author manuscript; Available in PMC 2024 Aug 16. (PMC11328916; doi:10.1016/j.cub.2024.01.025)
Supplement: MMC1 [file NIHMS1980531-supplement-MMC1.pdf]

Supplemental Information: Notes from Beethoven's genome

Laura W. Wesseldijk, Tara L. Henechowicz, David J. Baker, Giacomo Bignardi, Robert Karlsson, Reyna L. Gordon, Miriam A. Mosing, Fredrik Ullén and Simon E. Fisher

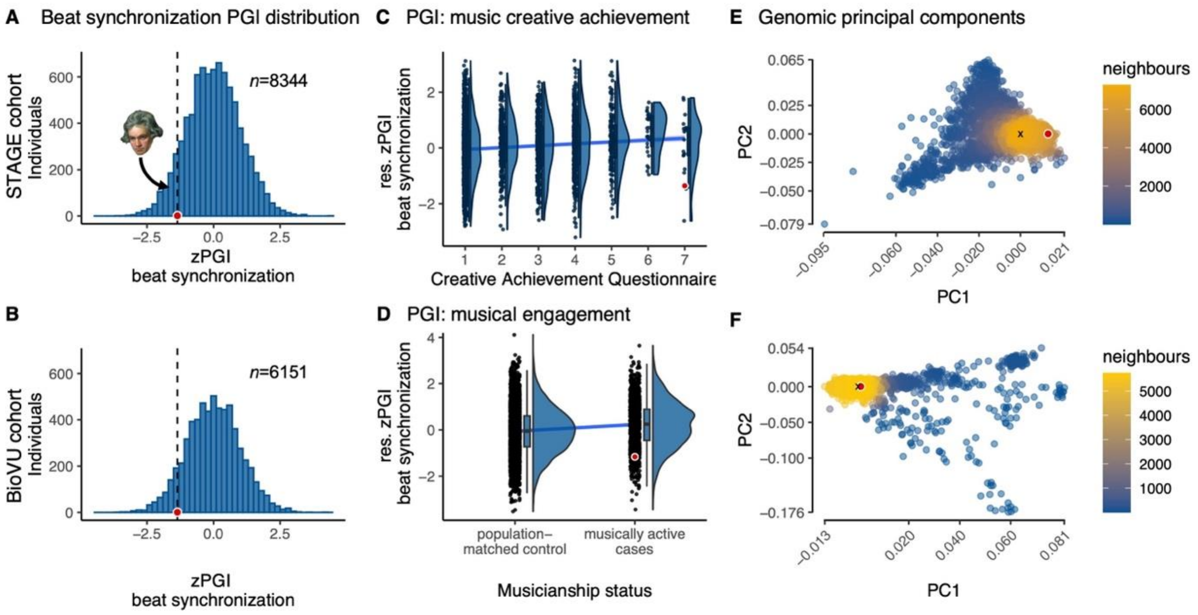

Figure S1. **Beethoven's beat synchronization PGI ranks between the 9th-to-11th percentile of modern samples' beat synchronization PGI.** (A–F) The distribution of the beat synchronization Polygenic Index (PGI) and Z-scores (zPGI) based on prior GWAS data (n=606,825)[S1]. Each bin includes individuals within a .2 PGI range. The black dashed line, and red dot represent Beethoven's PGI with respect to (A) STAGE[S2–5] and (B) BioVU[S1,S6] cohorts. (C) Raincloud plots[S7] depicting the relationship between the beat synchronization residualized (res.) PGI and Creative Achievement Questionnaire (CAQ; from levels 1: "I am not engaged in music at all" to 7: "I am professionally active as a musician and have been reviewed/featured in national or international media and have received an award for my musical activities") in STAGE. We first regressed the first 10 PCs from the PGIs and used the residuals for illustrative and analytical purposes. Dots represent STAGE individual scores; the red dot represents Beethoven's PGI; the oblique line represents the line of best fit, which was calculated excluding Beethoven's PGI. (D) Raincloud plots depicting the relationship between the beat synchronization residualized (res.) PGI and the musical engagement score in the electronic records from the BioVU health records[S6]. Individuals who were not identified as musically active are labelled as population-matched controls. Dots represent BioVU individual scores; the red dot represents Beethoven's PGI; the oblique line represents the line of best fit. Principal Component (PC) analyses were carried out for the two modern-day samples to generate ancestry covariates for comparison. Genomic PCs capturing genetic ancestry continuum are displayed for (E) STAGE and (F) BioVU. In red is Beethoven's location in the PC space. Each dot colour is informative about the density of neighbour points. Neighbours density was computed by using the R function ggpointdensity::geom_pointdensity[S8]. Given density, appropriate adjustment was set to one tenth of default bandwidth (adjust =.1). The black x represents the centre of the 2D PC space. PGI: Polygenic Index; STAGE:

Screening Twin Adults: Genes and Environment Swedish Twin Registry; BioVU: Vanderbilt Biorepository; PC: Principal Component; Res.: Residualized.

Supplemental experimental procedures

Participants - STAGE cohort

The Swedish Twin Registry includes The Study of Twin Adults: Genes and Environment (STAGE) cohort, consisting of approximately 32,000 adult twins born between 1959 and 1985[S2–S5]. In 2012 and 2013, 11,543 twins from this cohort completed a web survey on, among other things, music skills and music-related behaviour. In 2019 and 2020, individuals from this cohort, who provided saliva samples between 2006 and 2008, were genotyped. After quality control, genotype data were available for 8,343 individuals, of which 5,648 had also completed the web survey on music-related behaviour (aged between 27 and 54 years old, M = 40, SD = 7.8). The study and analyses of biomarkers were approved by the Regional Ethics Review Board in Stockholm (Dnr 2011/570-31/5, Dnr 2018/960-31/2, Dnr 2019-05879). The computations were enabled by resources provided by the National Academic Infrastructure for Supercomputing in Sweden (NAISS) and the Swedish National Infrastructure for Computing (SNIC) at Uppsala Multidisciplinary Center for Advanced Computational Science (UPPMAX) partially funded by the Swedish Research Council through grant agreements no. 2022-06725 and no. 2018-05973.

Genetic data processing and polygenic index calculation – STAGE cohort

Detailed information on the genetic data processing and quality control of the genotype data from the STAGE cohort can be found in Wesseldijk *et al.* [S9]. Whole genome sequence data from Ludwig van Beethoven were made publicly available[S10]. To combine the genotype data from Ludwig van Beethoven with the STAGE cohort, we first

extracted a list of 1,265,094 autosomal HapMap 3 CEU single-nucleotide polymorphisms (SNPs)[S11,S12], with imputation INFO score > 0.1 and minor allele frequency (MAF) ≥ 0.005 from the STAGE data. We then intersected this set with Ludwig van Beethoven's whole genome sequence data, restricted to sites in accessible regions, and with genotype quality ≥ 30. This resulted in a combined dataset with 981,614 HapMap 3 markers (78% of the HapMap3 markers originally available in STAGE). Genotype data were processed using bcftools (version 1.14) and PLINK 1.9 (version 1.90b4.9) and 2.0 (version 2.00a3.7LM)[S13,S14]. A principal component analysis (PCA) was performed in the merged sample (STAGE plus Ludwig van Beethoven) to generate ancestry covariates. PLINK was used to extract the first 20 principal components (PCs) based on common independent genotyped markers (MAF > 0.05, pairwise $R^2 < 0.1$). PCA was performed in unrelated individuals (identified by the KING algorithm in PLINK 2.0, with kinship cutoff 0.0442), and then projected in the full sample. Known long-range linkage disequilibrium (LD) regions (hg19 coordinates chr5:44-51.5 Mb, chr6:25-33.5 Mb, chr8:8-12 Mb, and chr11:45-57 Mb) were excluded from PCA calculations.

We generated polygenic indices (PGIs) based on summary statistics from a large genome-wide association study (GWAS) on self-reported beat synchronization ability in N = 606,825 individuals of European ancestry[S1]. The effect sizes were first re-estimated using the summary-data based on the best linear unbiased prediction (SBLUP) approach[S15,S16]. This approach computes effect sizes with best linear predictor properties that account for LD between SNPs. As a reference for LD, a random sample of 11,064 unrelated individuals was extracted from a set of 1,246,531 HapMap 3 SNPs that passed quality control in prior studies of the UK Biobank[S17]. PGIs were generated, based on these re-estimated effect sizes, for the 8,343 Swedish twin individuals in the STAGE

cohort plus Ludwig van Beethoven, using PLINK 1.9. This led to PGIs based on 910,648 SNPs, in contrast to 1,167,342 SNPs in the earlier published study by Wesseldijk *et al.*[S9] that validated the PGI for self-reported beat synchronization ability. We re-calculated associations between the PGIs based on 910,648 valid predictors and music-related outcomes, and this was barely different (maximum difference of 0.01 in beta estimates) from findings based on 1,167,342 predictors as reported in Wesseldijk *et al.*[S9].

Measures – STAGE cohort

"*Musical achievement*" was measured with an adapted and translated version of the Creative Achievement Questionnaire (CAQ)[S18–S21]. Using a seven-point scale, individuals rate their level of musical achievement, ranging from 1 'I am not engaged in music at all' via 4 'I have played or sung, or my music has been played in public concerts in my home town, but I have not been paid for this' to 7 'I am professionally active as a musician and have been reviewed/featured in national or international media and/or have received an award for my musical activities'.

Participants - BioVU cohort

The Vanderbilt BioVU sample includes 6,150 individuals (aged between 18 and 89 years old, M = 53, SD = 16.4) containing musically active cases and population matched controls whose data were extracted from the Synthetic Derivative (SD) database at the Vanderbilt University Medical Center (VUMC), approved by Vanderbilt Institutional Review Board for nonhuman subjects research (IRB #160302). Musically active cases (N = 1,259) were identified using an algorithmic search of 4 keywords and 449 regular expressions (selected examples include "musician", "vocalist", "songwriter", "drummer", "plays the piano", "playing the guitar", "played violin", "player of the cello", "plays saxophone", "flutist", "plays the flute", "player of oboe", "accordion player"; the full list is

given in supplementary table 2 of the phenotyping algorithm study by Niarchou *et al.*[S6]). Population matched controls (N = 4,891), retrieved from the same study, were matched for ethnicity, race, and median age at record length and did not have any of the music-related keywords/regular expressions[S1,S6].

Genetic data processing and PGI calculation – BioVU cohort

Detailed information on genetic data processing and quality control of the BioVU sample was reported in Niarchou *et al.*[S1]'s study. Niarchou *et al.*[S1] applied GWAS summary statistics on self-reported beat synchronization ability in N = 606,825 individuals of European ancestry to estimate beat synchronization PGIs in the BioVU target sample and to predict musically active case status (see section F of Supplementary Information in [S1]). The BioVU sample used for the previous PGI analysis included N = 6,152 individuals[S1], but we added an identity-by-descent (IBD) relatedness filter of 0.2 for this analysis which removed two related individuals yielding a final sample size of N = 6,150 for the present study.

To align the genotype data from Ludwig van Beethoven to the BioVU sample, we extracted a restricted set of 777,010 common, well-imputed 1000genomes SNPs from Ludwig van Beethoven's whole genome sequence data. This resulted in genotype data from 651,912 of the 1000genomes markers (83.9%) for Ludwig van Beethoven. We then extracted the genotype data for these 651,912 markers from the 6,150 individuals of the BioVU cohort and merged them with Ludwig van Beethoven's genotype data.

We carried out principal component analysis (PCA) using *FlashPCA* version 2.0[S22] in the merged sample (BioVU plus Ludwig van Beethoven) to generate 10 principal components. The sample used for PCA was preprocessed in PLINK1.9 for SNPs with MAF > 0.05, HWE p-value > 0.001, SNP missingness < 0.02, exclusion of SNPs in regions

with high LD in GRCH Build 37 (see list here: https://genome.sph.umich.edu/wiki/Regions_of_high_linkage_disequilibrium_(LD)), and chromosome 17 inversion region (chr17:40-45Mb), and filtering out strand ambiguous and multi-allelic SNPs. Next, the data underwent two rounds of LD pruning at $r2 < 0.2$ in 200kb SNP windows.

PGIs were estimated following the methods outlined in Niarchou *et al.*[S1]. Namely, the effect sizes were first re-estimated using PRS-CS-auto and the 1000genomes European LD reference panel as the initial beat synchronization PGI calculation using the BioVU sample in Niarchou *et al.*[S1]'s study. PRS-CS-auto uses a Bayesian regression framework and places a continuous shrinkage prior on SNP effect sizes, then tunes the phi parameter automatically[S23]. Next, we generated PGIs based on summary statistics from the large GWAS on self-reported beat synchronization ability in N = 606,825 individuals of European ancestry. PGIs were generated, based on these re-estimated effect sizes, for 6,150 individuals in the BioVU cohort plus Ludwig van Beethoven, using PLINK 1.9[S13,S14]. This led to PGIs based on 651,912 SNPs in contrast to 777,010 SNPs in Niarchou *et al.* [S1] beat synchronization PGI. In the Niarchou *et al.*[S1] PGI analysis with N = 6,152 individuals, PGIs for beat synchronization were significantly associated with musical engagement (OR = 1.34 per s.d. increase in PGI; $p < 2 \times 10^{-16}$; Nagelkerke's $R^2 = 2\%$; 95% CI, (1.26, 1.43)). Results recalculated with 651,912 valid predictors in the sample of the combined BioVU cohort plus Beethoven sample (N = 6,151) remained significant (OR = 1.35 per s.d. increase in PGI; $p < 2 \times 10^{-16}$; Nagelkerke's $R^2 = 2\%$; 95% CI, (1.27, 1.44)).

Full Acknowledgements: We thank the Swedish twins, individuals in the BioVU cohort and the participants of 23andMe, Inc. for their participation and the Swedish Twin Registry, Vanderbilt's BioVU and employees of 23andMe for making data available for this study. We thank Tristan James Alexander Begg for sharing the genome sequence data from Ludwig van Beethoven and Abdel Abdellaoui for his valuable advice. The Swedish Twin Registry is managed by Karolinska Institutet and receives funding through the Swedish Research Council under the Grant No. 2017-00641. The computations were enabled by resources provided by the National Academic Infrastructure for Supercomputing in Sweden (NAISS) and the Swedish National Infrastructure for Computing (SNIC) at Uppsala Multidisciplinary Center for Advanced Computational Science (UPPMAX) partially funded by the Swedish Research Council through grant agreements no. 2022-06725 and no. 2018-05973. Vanderbilt University Medical Center's BioVU projects are supported by numerous sources: institutional funding, private agencies, and federal grants. These include NIH funded Shared Instrumentation Grant S10OD017985, S10RR025141, and S10OD025092; CTSA grants UL1TR002243, UL1TR000445, and UL1RR024975. Genomic data are also supported by investigator-led projects that include U01HG004798, R01NS032830, RC2GM092618, P50GM115305, U01HG006378, U19HL065962, R01HD074711, K07CA172294, 14GRNT20460090, P01DK038226, R24DK96527, U01HG004798, R01LM010685, R01NS032830, R01EY012118, K12HD043483, R01DK078616, RC2GM092618, APP1064524, R01CA162433, P01HL056693, P50GM115305, U01HG006378, U19HL065962, U01HG004603, P50CA09813, R01HD074711, R03HD078567, R01DK080007, and P50HL081009. S.E.F., F.U., M.A.M., L.W.W. and G.B. acknowledge support of the Max Planck Society. G.B. also acknowledges funds from the German Federal Ministry of Education and Research (BMBF). T.L.H. is supported by the Data Science Institute at the University of Toronto and CANSSI Ontario STAGE.

Author contributions: Conceptualization, L.W.W., T.L.H., D.J.B., G.B. and S.E.F.; Formal analyses, L.W.W., T.L.H. and R.K.; Visualization, G.B.; Writing – Original Draft, L.W.W., D.J.B. and S.E.F.; Writing – Review & Editing, L.W.W., T.L.H., D.J.B., G.B., R.K., R.L.G., M.A.M., F.U. and S.E.F..; Funding Acquisition, R.L.G., F.U. and S.E.F.

Supplemental references

S1. Niarchou, M., Gustavson, D.E., Sathirapongsasuti, J.F., Anglada-Tort, M., Eising, E., Bell, E., McArthur, E., Straub, P., Aslibekyan, S., Auton, A., et al. (2022). Genome-wide association study of musical beat synchronization demonstrates high polygenicity. Nat. Hum. Beh. 6, 1292-1309. 10.1038/s41562-022-01359-x.

S2. Lichtenstein, P., De Faire, U., Floderus, B., Svartengren, M., Svedberg, P., and Pedersen, N.L. (2002). The Swedish Twin Registry: a unique resource for clinical, epidemiological and genetic studies. J. Intern. Med. 252, 184-205. 10.1046/j.1365-2796.2002.01032.x.

S3. Lichtenstein, P., Sullivan, P.F., Cnattingius, S., Gatz, M., Johansson, S., Carlstrom, E., Bjork, C., Svartengren, M., Wolk, A., Klareskog, L., et al. (2006). The Swedish Twin Registry in the third millennium: an update. Twin Res. Hum. Genet. 9, 875-882. 10.1375/183242706779462444.

S4. Magnusson, P.K., Almqvist, C., Rahman, I., Ganna, A., Viktorin, A., Walum, H., Halldner, L., Lundstrom, S., Ullen, F., Langstrom, N., et al. (2013). The Swedish Twin Registry: establishment of a biobank and other recent developments. Twin Res. Hum. Genet. 16, 317-329. 10.1017/thg.2012.104.
S5. Kyaga, S., Lichtenstein, P., Boman, M., Hultman, C., Långström, N., and Landén, M. (2011). Creativity and mental disorder: family study of 300,000 people with severe mental disorder. Br. J. Psych. 199, 373-379. 10.1192/bjp.bp.110.085316.
S6. Niarchou, M., Lin, G., Lense, M.D., Gordon, R., and Davis, L. (2021). Medical phenome of musicians: An investigation of health records collected on 9803 musically active individual. Ann. NY Acad. Sci. 1505(1), 156-168. 10.1111/nyas.14671.
S7. Allen, M., Poggiali, D., Whitaker, K., Marshall, T.R., and Kievit, R.A. (2019). Raincloud plots: a multi-platform tool for robust data visualization. Wellcome Open Res. 4, 63. 10.12688/wellcomeopenres.15191.1.
S8. Kremer, L.P.M., and Anders, S. (2019). ggpointdensity: A Cross Between a 2D Density Plot and a Scatter Plot. Version 0.1.0.
S9. Wesseldijk, L.W., Abdellaoui, A., Gordon, R.L., Ullén, F., and Mosing, M.A. (2022). Using a polygenic score in a family design to understand genetic influences on musicality. Sci. Rep. 12, 14658. 10.1038/s41598-022-18703-w.
S10. Begg, T.J.A., Schmidt, A., Kocher, A., Larmuseau, M.H.D., Runfeldt, G., Maier, P.A., Wilson, J.D., Barquera, R., Maj, C., Szolek, A., et al. (2023). Genomic analyses of hair from Ludwig van Beethoven. Curr. Biol. 33, 1431-1447.e1422. 10.1016/j.cub.2023.02.041.
S11. Altshuler, D.M., Gibbs, R.A., Peltonen, L., Altshuler, D.M., Gibbs, R.A., Peltonen, L., Dermitzakis, E., Schaffner, S.F., Yu, F., Peltonen, L., et al. (2010). Integrating common and rare genetic variation in diverse human populations. Nature 467, 52-58. 10.1038/nature09298.
S12. Bulik-Sullivan, B.K., Loh, P.R., Finucane, H.K., Ripke, S., Yang, J., Schizophrenia Working Group of the Psychiatric Genomics, C., Patterson, N., Daly, M.J., Price, A.L., and Neale, B.M. (2015). LD Score regression distinguishes confounding from polygenicity in genome-wide association studies. Nat. Genet. 47, 291-295. 10.1038/ng.3211.
S13. Chang, C.C., Chow, C.C., Tellier, L.C., Vattikuti, S., Purcell, S.M., and Lee, J.J. (2015). Second-generation PLINK: rising to the challenge of larger and richer datasets. Gigascience 4, 7. 10.1186/s13742-015-0047-8.
S14. Purcell, S.M., and Chang, C.C. (2007). PLINK 2.0.
S15. Robinson, M.R., Kleinman, A., Graff, M., Vinkhuyzen, A.A.E., Couper, D., Miller, M.B., Peyrot, W.J., Abdellaoui, A., Zietsch, B.P., Nolte, I.M., et al. (2017). Genetic evidence of assortative mating in humans. Nat. Hum. Beh. 1, 0016. 10.1038/s41562-016-0016.
S16. Ni, G., Zeng, J., Revez, J.R., Wang, Y., Ge, T., Restaudi, R., Kiewa, J., Nyholt, D.R., Coleman, J.R.I., Smoller, J.W., et al. (2021). A Comparison of Ten Polygenic Score Methods for Psychiatric Disorders Applied Across Multiple Cohorts. Schizophrenia Working Group of the Psychiatric Genomics Consortium; Yang J, Visscher PM, Wray NR. Biol. Psychiatry 90, 611-620 doi: 10.1016/j.biopsych.2021.04.018.
S17. Abdellaoui, A., Dolan, C.V., Verweij, K.J.H. and Nivard, M.G. (2022). Gene–environment correlations across geographic regions affect genome-wide

association studies. Nat. Genet. 54, 1345–1354 https://doi.org/10.1038/s41588-022-01158-0
S18. Wesseldijk, L.W., Mosing, M.A., and Ullén, F. (2019). Gene-environment interaction in expertise: The importance of childhood environment for musical achievement. Dev. Psychol. 10.1037/dev0000726.
S19. Carson, S.H., Peterson, J.B., and Higgins, D.M. (2005). Reliability, Validity, and Factor Structure of the Creative Achievement Questionnaire. Creat. Res. J. 17, 37-50. 10.1207/s15326934crj1701_4.
S20. Mosing, M.A., Verweij, K.J., Abe, C., de Manzano, O., and Ullen, F. (2016). On the Relationship Between Domain-Specific Creative Achievement and Sexual Orientation in Swedish Twins. Arch. Sex. Behav. 45, 1799-1806. 10.1007/s10508-016-0708-4.
S21. Mosing, M.A., Verweij, K.J.H., Madison, G., Pedersen, N.L., Zietsch, B.P., and Ullén, F. (2015). Did sexual selection shape human music? Testing predictions from the sexual selection hypothesis of music evolution using a large genetically informative sample of over 10,000 twins. Evol. Hum. Behav. 36, 359-366. https://doi.org/10.1016/j.evolhumbehav.2015.02.004.
S22. Abraham, G., & Inouye, M. (2014). Fast principal component analysis of large-scale genome-wide data. PloS one, 9(4), e93766. https://doi.org/10.1371/journal.pone.0093766
S23. Ge, T., Chen, C.-Y., Ni, Y., Feng, Y.-C.A., and Smoller, J.W. (2019). Polygenic prediction via Bayesian regression and continuous shrinkage priors. Nat. Comm. 10, 1776. 10.1038/s41467-019-09718-5.
